# Proteome Profiling of Membrane-Free Stem Cell Components by Nano-LS/MS Analysis and Its Anti-Inflammatory Activity

**DOI:** 10.1155/2019/4683272

**Published:** 2019-11-04

**Authors:** Venu Venkatarame Gowda Saralamma, Preethi Vetrivel, Seong Min Kim, Sang Eun Ha, Ho Jeong Lee, Sang Joon Lee, Young Sil Kim, Jung Eun Pak, Hye Jin Lee, Jeong Doo Heo, Gon Sup Kim

**Affiliations:** ^1^Research Institute of Life Science and College of Veterinary Medicine, Gyeongsang National University, 501 Jinju-daero, Jinju, Gyeongnam 52828, Republic of Korea; ^2^Gyeongnam Department of Environment Toxicology and Chemistry, Toxicity Screening Research Center, Korea Institute of Toxicology, 17 Jegok-gil, Munsan-eup, Jinju, Gyeongnam 52834, Republic of Korea; ^3^T-Stem Co., Ltd., Changwon, Gyeongnam 51573, Republic of Korea

## Abstract

The use of adipose-derived stem cells (ADSCs) to enhance wound healing and tissue regeneration is progressively being accepted. Proteomic profiling of cultured ADSCs by mass spectrometry (MS) is a valuable tool to determine the identity of the proteins involved in multiple pathways, which make these ADSCs unique. In the current study, Nano-LC-MS/MS analysis was implemented on the membrane-free stem cell component (MFSCC), and the MS analysis revealed the presence of 252 proteins, that are involved in several biological functions, like metabolic process, biological regulation, developmental process, cell proliferation, and many more. Furthermore, bioinformatic analyses of the identified proteins in MFSCC found them to be involved in versatile pathways, like integrin pathway and wound healing response-related pathways. In addition, we also investigated the anti-inflammatory effects of MFSCC on lipopolysaccharide (LPS) stimulated mouse macrophage (RAW264.7) cells. The cell cytotoxicity of MFSCC was measured using MTT and LDH assays, the production of nitric oxide (NO) was measured by the Griess assay, and the protein expression levels of inducible nitric oxide (iNOS) and cyclooxygenase (COX-2) were examined by western blot analysis. The results showed that MFSCC concentrations ranging from 0.1 to 3 *μ*g/mL did not show any significant cytotoxicity in LPS-induced RAW264.7 cells. Treatment with MFSCC of LPS-stimulated RAW264.7 cells significantly suppressed the production of NO and the expression of iNOS and COX-2 proteins related to inflammation. The present findings lead to a better understanding of the therapeutic potential of MFSCC and strongly promote it for the future clinical development of novel non-cell-based stem cell therapeutics.

## 1. Introduction

Adult stem cells are kinds of cell that offer promise in regenerative medicine for cell-based therapies [1]. Human adipose tissue has been introduced as a replacement source for multipotent stem cells, and therefore, adipose tissue-derived stem cells (ADSCs) are considered to be optimal for utilization in regenerative therapies, wound healing, and cardiovascular disorders treatments [2, 3]. ADSCs demonstrate advancement over mesenchymal stem cells derived from alternative sources like bone marrow, in that they can be repeatably and conveniently harvested using simple invasive techniques with low morbidity. Their secretion of equatorial factors imposes the regenerative and therapeutic outcomes in an excessively wide selection of applications [2, 4]. ADSCs are also known for their powerful anti-inflammatory and angiogenic properties, and ADSCs have been examined as a means to aid the wound healing process [5, 6]. These cells function in a paracrine manner, stimulating the surrounding cells and promoting angiogenesis [7]. Taken together, these explicit characteristic features of ADSCs make them extremely compatible with clinical applications, and thus, the therapeutic potential of ADSCs is tremendous. Basic research on cell-mediated therapies shows significant promise to use them as therapeutic tools, whereas cell-based therapy faces several obstacles, such as the cells being difficult to grow, preserve, and transport, before being administered to the patient. Sufficient evidence indicates that stem cells exert their effects predominantly through the membrane-based cell-to-cell interaction and secretion of regenerative factors in the injured cells. Meanwhile, studies have also shown that stem-cell conditioned media (CMs) have possible therapeutic applications in neural, myocardial, and wound healing [8]. The use of secretome containing CM has distinct advantages compared to the use of stem cells, as CM can be manufactured, freeze-dried, packaged, and transported more conveniently. Research has shown that a synthetic cell-mimicking microparticle can be fabricated that recapitulates stem cell functions in tissue repair [4]. More importantly, this cell-free therapeutic approach can be altered for safety and dosage, like a normal pharmaceutical agent. Based on the above strands of evidence, we have here for the first time made an effort to prepare membrane-free stem cell components (MFSCCs), considering them as an alternative source for a therapeutic approach to overcome the limitation of cell-based therapies. We hypothesize that MFSCC can exert similar regenerative outcomes as real stem cells, and that they may be superior, since they are more stable during storage, and do not stimulate immune reaction, since they are not real cells.

Protein-based pharmaceuticals have made significant advances during the past decade. The use of peptide and protein-derived drugs has traditional problems with other pharmaceuticals due to issues with bodily distribution, drug administration (oral), and immunogenicity issues, e.g., due to the complication of preparation [9]. The currently available technologies and knowledge dealing with immunogenicity and immunotoxicity and the advancement in the formulation methodologies, protein engineering, administration routes, and production techniques have now positioned protein-based biopharmaceuticals firmly in the Pharma pipelines [10]. Proteomics is one such vital technique to interpret the biological processes since proteins accomplish all the functions that are accomplished by proteins in the cell [11]. Proteomic technologies will be an exceptional tool for the quantitation and global detection of proteins, which will create new opportunities and challenges for those who are seeking to gain greater empathy for the diseases. The progressive bioinformatics combined with high-throughput proteomic technologies is comprehensively implemented to identify the molecular signatures of protein pathways and disease-promoting signaling cascades [12]. In proteomics, mass spectrometry plays a significant role and has become an essential tool for molecular and cellular biology. Mining low abundance proteins and the integration of proteomics with genomics and metabolomics data in cells have become challenging tasks and remain to be solved. Nanoscale liquid chromatography coupled to tandem mass spectrometry (Nano-LC-MS/MS) has become an important tool in the field of proteomics, and its sensitivity has predominated over the typical LC-MS/MS that permits the analysis of peptide mixtures in sample-limited conditions [13]. Proteome mapping serves as a starting point for establishing a comprehensive database of stem cells proteome in several studies for a better understanding of its molecular action. Therefore, protein profiling of MFSCC may contribute to gaining the novel biological knowledge with respect to the therapeutic potential of noncell-based stem cell therapeutics, along with the probability of identifying potential therapeutic targets.

In the present study, we report for the first time the preparation of membrane-free stem cell components (MFSCCs) and performed proteome profiling by implementing Nano LC-MS/MS coupled with bioinformatics tools to study the entire spectrum of paracrine factors present in MFSCCs. We also examined the anti-inflammatory effect of MFSCC on lipopolysaccharide (LPS) stimulated RAW246.7 cells to know its therapeutic ability.

## 2. Materials and Methods

### 2.1. Preparation of Membrane-Free Stem Cell Components (MFSCCs)

**Figure 1:**
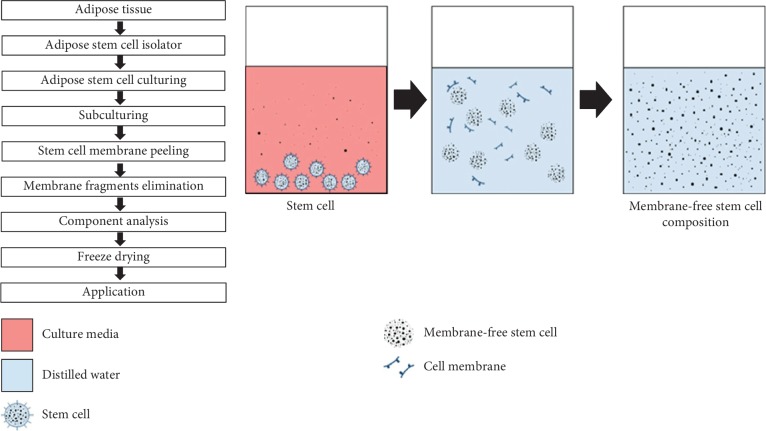
Schematic diagram showing the workflow of membrane-free stem cell component (MFSCC) preparation and conceptual illustration showing the process of MFSCC preparation.

The MFSCCs used in this study were produced with the patented technology and are compositions of stem cell components, where the membranes of stem cells were removed, after being separated and cultured from human body fat tissue. In brief, the fat tissue was provided by a healthy female in her twenties with BMI (25∼29.9) obesity (2-degree obesity), which after the blood tests and doctor's diagnosis was proven to be appropriate. The blood tests conducted were hepatitis B virus (HVB), hepatitis C virus (HCV), human T lymphocytic virus (HTLV), human immunodeficiency virus (HIV), parvovirus B19, cytomegalovirus (CMV), Epstein–Barr virus (EBV), and Treponema pallidum. The donor gave written informed consent, and the Regional Committee on Biomedical Research Ethics approved the clinical protocol. Fat tissues, which have been proven to be safe through the prescreenings mentioned above, were separated and purified, and the extracted cells were cultured in a serum-free cell culture medium at 37°C and 5% CO_2_, in a standard incubator. After the cell growth reached (70–80)% confluence, the cells were subcultured until 6–8 passages. A certain amount of stem cells were collected, the cell membranes were removed by ultrasonication, and the debris was eliminated from the membranes by centrifugation at 800–1500*g*, following successive filtration. The aqueous solution of MFSCC was further lyophilized, made into powder form, and stored. MFSCC, the final product, is proven to be a nontoxic substance through nine safety tests at the Good Laboratory Practice (GLP) accreditation authority.  Figure 1 shows the schematics of the process of MFSCC preparation.

### 2.2. Preparation of Protein Sample

MFSCC was dissolved in the RIPA buffer containing protease and phosphatase inhibitor and subsequently sonicated to ensure complete lysis. Protein samples were then quantified using the Pierce™ BCA protein assay kit (Thermo Scientific™, Waltham, MA, USA), following the manufacturer's protocol, and stored at −80°C until further analysis.

### 2.3. One-Dimensional Gel Electrophoresis

**Figure 2:**
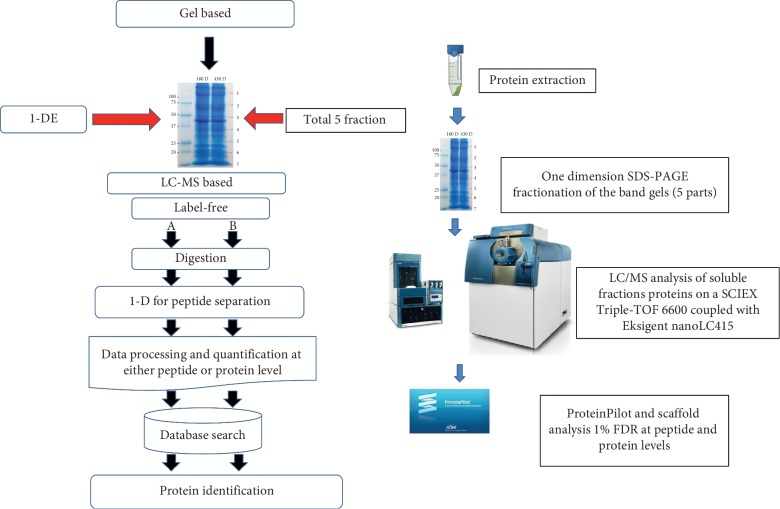
Schematic showing the proteome profiling of membrane-free stem cell components by LS/MS analysis.

For the proteome analysis of MFSCC (Figure 2), a total of 25 *μ*L of protein lysate in the RIPA buffer containing protease and phosphatase inhibitor was mixed with reducing sample buffer. 20 *μ*g of total protein was diluted with the denaturing sample buffer (10% SDS, 20% glycerol, 0.5 M Tris-HCl pH 8.8, 1% bromophenol blue, and 0.2% DTT) and heated at 95°C for 5 min, and the same samples were considered for one-dimensional SDS-PAGE. 12% SDS-PAGE gels were used for the protein sample separation in one-dimensional gel electrophoresis. Electrophoresis was conducted at 100 V for 60 min, followed by at 200 V for 2 h. Coomassie Brilliant R250 (Sigma-Aldrich, ST. Louis, MO, USA) stain was used for staining the electrophoresed gels, and destaining was done with water.

### 2.4. In-Gel Digestion for Mass Spectrometric Analysis

SDS-PAGE gel lane containing all protein bands from the one-dimensional gel was excised using a razor blade from top to bottom, and the excised gel slices were washed twice with 100 *μ*L of distilled water for 15 min at room temperature (RT). Excised gel bands were destained with 50% acetonitrile and shrunk with 100% acetonitrile. Then, 500 *μ*L of 50 mM ammonium bicarbonate was used for 5 min at room temperature (RT), to soak the proteins in gel. Next, 500 *μ*L acetonitrile was added to replace the ammonium bicarbonate solution by pipetting and incubated for 5 min at RT. After acetonitrile incubation, the gel slices were vacuum-dried completely. The gel slices were then incubated in 10 mM DTT/0.1 M ammonium bicarbonate for 45 min at 56°C, followed by alkylation by incubation with 55 mM iodoacetamide/0.1 M ammonium bicarbonate for 30 min at RT in the dark. After alkylation, the gel slices were dried again, and dried gel slices were inflated in 5 *μ*L of digestion buffer that contained 0.1% *n*-octyl glucoside, 50 ng/mL of sequencing grade trypsin (Promega, Madison, MI, USA), and 25 mM ammonium bicarbonate for rehydration. After rehydration, the band slices were incubated overnight at 37°C in 50 *μ*L digestion buffer (without trypsin), to allow enzymatic cleavage in the silicon zed tube. For each gel band, about 0.1 *μ*g of the enzyme was used. Peptides were extracted from the gel slices with 33% water, 66% acetonitrile, and 0.1% trifluoroacetic acid (TFA). After centrifugation, the peptides were transferred into a fresh tube and speed vacuum-dried with a speed vacuum lyophilizer (Hanil, Korea). The dried peptides from the gel slices were stored at −80°C, before analysis.

### 2.5. Nanoflow Liquid Chromatography

On redisolving, dried peptides were extracted from the gel slices in 20 *μ*L of 5% formic acid and analyzed on-line by Nanoflow LC-MS/MS. Eksigent Nano-LC 415 system (EKsigent, Dublin, USA) was used to performing all Nano-LC-MS/MS experiments and was connected to TripleTOF 6600 mass spectrometry system (SCIEX, Redwood City, CA, USA) with a nanoelectrospray ion source (New Objective, Woburn, MA, USA). The tryptic digested peptides were separated in a 15 cm analytical column (ChromXP C18, 75 *μ*m × 15 cm, 3 *μ*m, 120 Å, EKsigent, Dublin, USA) with a 90 min gradient from 5 to 60 % acetonitrile in 0.1% formic acid. The effluent from nano-LC was precisely electrosprayed into a mass spectrometer.

### 2.6. LC-MS/MS Analysis

For gel-free proteomic analysis, protein samples were resuspended in water/formic acid solution (water in 5% formic acid). Online Nano-HPLC was conducted by using an Eksigent nano-LC415 system (EKsigent, Dublin, USA). A C18 nano-LC trap column (ChromXP, 350 *μ*m × 0.5 mm, 3 *μ*m, 150 Å) was used, and the samples were loaded and washed with Nano-HPLC buffer A at a rate of 300 nL/min for 10 min. An elution gradient of 5–60% water (0.1% formic acid) over a 90 min gradient period was used on an analytical ChromXP C18 column (75 *μ*m × 15 cm, 3 *μ*m, 120 Å) with a nanospray tip. Data acquisition was executed with a triple time-of-flight (TOF) 6600 system (SCIEX, Redwood City, CA, USA) coupled with the nanospray source (New Objective, Woburn, MA, USA), with a pulled 10 *μ*m fused silica emitter, 360/20 *μ*m (New Objective, Woburn, MA, USA). Data were acquired using an ion-spray voltage floating (ISVF) of 2.3 kV, curtain gas of 28 PSI, ion source gas (GS1) of 15 PSI, and interface heater temperature of 150°C. For information-dependent acquisition (IDA), the survey scans range was set between 250 and 2000 *m*/*z* (250 ms accumulation time), followed by a dependent MS/MS scan with a mass range set between 100 and 2000 *m*/*z* (100 ms accumulation time) of the 20 most intense ions in the high sensitivity mode with a 2+ to 5+ charge state. Mass tolerance was for a period of 50 ppm, and dynamic exclusion was for a period of 15 s. The rolling collision energy was implemented.

### 2.7. MS Data Processing

Data files were processed after the MS/MS analysis using UniProt and Protein Pilot 5.0.1 (SCIEX, Redwood City, CA, USA) database software. Proteins were successfully identified based on the combined MS and MS/MS spectra, at 95% or higher confidence spell, using their scores in the MASCOT V2.5 search engine (Matrix Science Ltd, London, UK) with the following search parameters: Brassica database, single missed cleavage sites, trypsin as the digestion enzyme, fixed modifications of carbamidomethyl (C) and oxidation of methionine, ±0.1 Da precursor ion tolerance, and ±0.1 Da MS/MS fragment ion tolerance.

### 2.8. Criteria for the Identification of Proteins

The identification of protein was performed using the software ProteinPilot 5.0.1 that follows the Paragon algorithm. A detailed search was undertaken with the following defined parameters like iodoacetamide modified by cysteine alkylation and digestive enzyme trypsin. A search on the Tandem mass spectrometric data was carried out against the database SwissProt (version 2018/02) and the Brassica peptide sequences (downloaded on August 2017; 209326 sequences in total). The results obtained from the search were manually curated to get the identified proteins with the help of a 1% global false discovery rate (FDR) value determined by the ProteinPilot software and Scaffold (version Scaffold_4.8.4, Proteome Software Inc., Poland, OR). The data were considered to validate the MS/MS-based peptide and identification of proteins.

### 2.9. Bioinformatic Analysis of the Obtained Proteins

Identified proteins from MS/MS analysis were further submitted to Web Gestalt (http://www.webgestalt.org), a web-based gene ontology (GO) tool to find the GO annotations of the obtained proteins. The enriched GO was obtained in terms of biological process, cellular component, and molecular function. Identified proteins were also subjected to pathway analysis by utilizing the PANTHER database (http://www.pantherdb.org). The potential protein-protein interactions of selected genes were investigated using STRING (Search Tool for the Retrieval of Interacting Genes) database version: 10.5 (https://string-db.org). To display protein interactions, selected proteins were uploaded into STRING database and evaluated using Cytoscape Software version Cytoscape_v3.7.1 (https://www.cytoscape.org). String is an online tool that aids in providing unambiguous comprehensive coverage and also helps to access the interaction of the experimental data.

### 2.10. Cell Culture and Cell Viability Assay

The mouse macrophages RAW246.7 cells were obtained from the American Type Culture Collection (ATCC) (USA) and cultured in complete Dulbecco's Modified Eagle Medium (DMEM) containing 10% heat-inactivated FBS with antibiotics 1% penicillin/streptomycin. The cells were grown and maintained by incubating at 37°C in a humidified atmosphere of 5% CO_2_. Cells were seeded at a density of 1 × 10^5^ cells/mL in a 48-well plate and incubated overnight. After the cells were grown to optimal confluence, cells were either treated with different concentrations of MFSCC (0.1 to 3 *μ*g/mL) alone or pretreated with LPS (1 *μ*g/mL). They were then incubated for 24 h; 3-(4,5-dimethylthiazol-2-yl)-2,5-diphenyltetrazolium bromide (MTT) which was obtained from Duchefa Biochemie (Haarlem, the Netherlands) (25 *μ*L; 5 mg/mL) was added to each well, and the cells then incubated at 37°C for about 4 h. Furthermore, the medium was removed completely, and the insoluble formazan crystals formed were dissolved in DMSO for 30 min. The absorbance was measured at a wavelength of 540 nm using a microplate reader (BioTek, Winooski, VT, USA). Cell viability was expressed as a percentage of proliferation versus the untreated group of cells.

For lactate dehydrogenase (LDH) assay, a cell cytotoxicity assay kit (Duzen Bio Co., Ltd., Guro-gu, Republic of Korea) was used according to the manufacturer's instructions. In brief, the culture medium of RAW246.7 cells after treatment with different concentrations of MFSCC (0.1 to 3 *μ*g/mL) was transferred into a 96-well plate and mixed with 100 *μ*L of the working solution. The mixture was incubated in RT for 30 min in the dark. The absorbance of the mixture was then measured using a microplate reader (BioTek, Winooski, VT, USA) at 450 nm, which reflects the level of LDH released into the medium.

### 2.11. NO Detection Assay

Griess assays were implemented to measure NO production in the cultured cells. RAW264.7 cells were cultured at a density of 5 × 10^4^ cells/mL in 96-well plates with or without LPS pretreatment for 30 min and then treated with the indicated concentration of MFSCC (0.4, 0.8, 1, 1.5, and 2 *μ*g/mL) and incubated for 24 h. After incubation, the supernatants in each group were collected and mixed with 50 *μ*L of Griess reagent for 10 min at RT. The nitrate concentration was measured at 520 nm using a microplate reader. To generate the standard curve, sodium nitrite (NaNO_2_) was used and the NO production in the culture medium was estimated by the NO_2_ concentration.

### 2.12. Western Blot Analysis of COX-2 and iNOS Proteins

RAW264.7 cells were seeded in 6-well plates, stimulated with LPS, cotreated with the indicated concentration of MFSCC (0, 1, and 2 *μ*g/mL), and incubated for 24 h at 37°C, and the cells were lysed in ice-cold RIPA buffer (50 mM Tris-HCl (pH 8.0), 0.5% sodium deoxycholate, 1 mM EDTA, 150 mM NaCl, 0.1 sodium dodecyl sulfate (SDS), and 1% NP-40). The protein concentration was determined using the Pierce™ BCA protein assay kit (Thermo Scientific™, Waltham, MA, USA), in accordance with the manufacturer's protocol. About 10 *μ*g of protein was separated with 10% SDS-PAGE and transferred onto a PVDF membrane using the TE 77 Semi-Dry Transfer Unit (GE Healthcare Life Sciences, Buckinghamshire, UK). The blots were then blocked with 5% skimmed milk or with 5% BSA for 1 h at RT and further incubated with primary antibodies at 1 : 1,000 dilutions for overnight (antibodies to COX-2 and iNOS were obtained from Santa Cruz Biotechnology (Santa Cruz, CA, USA)). The membranes were washed with the TBST buffer for 5 times in 10 min intervals and then probed with their appropriate horseradish peroxidase-coupled secondary antibody for 3 h at RT. The blots were visualized using Clarity™ ECL substrate reagent (Bio-Rad, Hercules, CA, USA) and quantified by densitometry analysis using the Image J (http://rsb.info.nih.gov) program. The densitometry readings of the bands were normalized to the expression of loading control *β*-actin. The experiment was repeated three times to get concordant results.

### 2.13. Statistical Analysis

The data were expressed as mean ± standard deviation (SD) and analyzed by SPSS (10.0) for statistical significance using one-way analysis of variance (ANOVA). *p* < 0.05 was considered as statistically significant.

## 3. Results

### 3.1. Nano-LC-MS/MS Analysis and Data Processing of MFSCC Proteins

The entire protein profiling of MFSCC was achieved by Nano-LC-MS/MS analysis by preprocessing by the database UniProt and ProteinPilot 5.0.1 (SCIEX, Redwood City, CA, USA) software. By the combination of MS and MS/MS spectra, the proteins with 95% and higher confidence interval were identified using the score from MASCOT V2.5 search engine (Matrix Science Ltd, London, UK). With the search parameters, Brassica database, digestive enzyme trypsin, single missed cleavage sites, modifications of carbamidomethyl (C) and oxidation of methionine, ±0.1 Da precursor ion tolerance, and ±0.1 Da MS/MS fragment ion tolerance were used. The search results were manually curated to obtain proteins using a 1% global false discovery rate (FDR) determined by the software ProteinPilot and Scaffold (Version Scaffold_4.8.4, Proteome Software Inc., Portland, OR) and used in the validation of the MS/MS-based peptide and proteins that were identified. About 252 proteins were identified successfully with the above criteria from the MFSCC with 1% FDR value ([Supplementary-material supplementary-material-1] of the Supplementary Information (SI)).

### 3.2. Functional Enrichment Analysis of Identified Proteins in MFSCC

**Figure 3:**
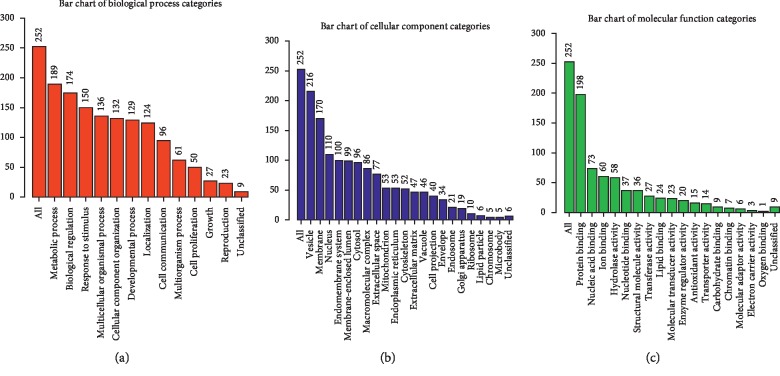
Gene ontology analysis of MFSCC proteins. Bar diagram of the significantly enriched GO terms of 252 proteins from MFSCC. Three bar diagrams showing the Hub genes involved in gene ontology in terms of (a) biological process, (b) cellular component, and (c) molecular function are depicted (http://www.webgestalt.org).

**Figure 4:**
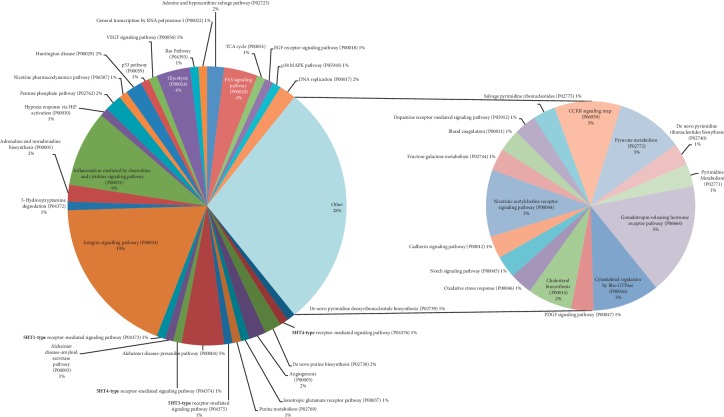
Pathway enrichment of the obtained proteins from MFSCC that are grouped based on the enriched pathway using the PANTHER database. % of proteins participating in each pathway is represented by a PIE chart (a representative PIE chart of the output obtained from PANTHER analysis).

**Figure 5:**
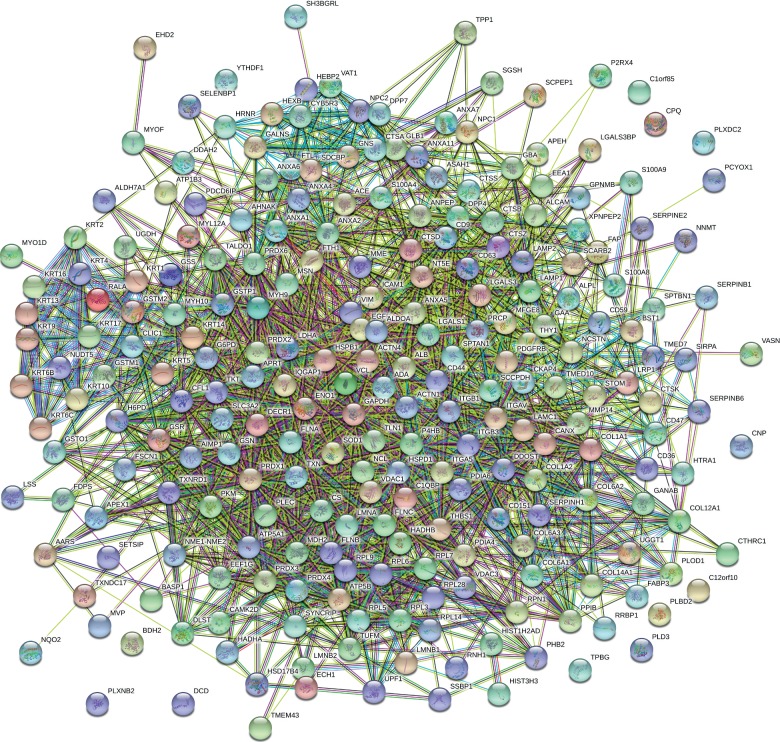
STRING analysis of the total proteins from membrane-free stem cell components (MFSCCs). For the protein-protein interaction of the 252 proteins from MFSCC, STRING database, version 10.5 (http://string-db.org), was used. Interactions were estimated with a high confidence level of 0.400 and were included in the analyses.

**Figure 6:**
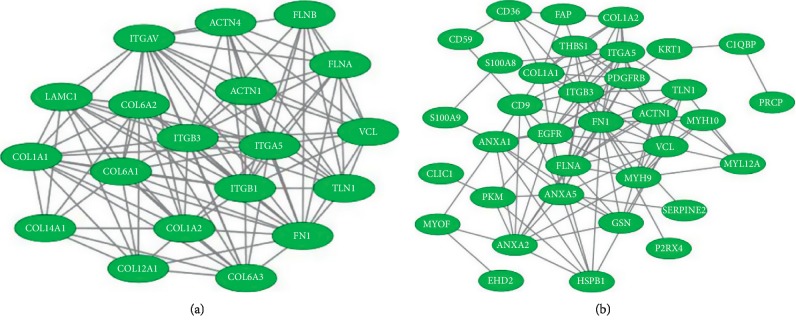
STRING analysis of integrin signaling pathways and response to wound healing pathways. To determine the protein-protein interactions of the (a) 19 proteins among a total of 252 proteins from the proteome profiling of MFSCC, which are integrin signaling proteins and (b) 36 proteins among a total of 252 proteins, which are response to wound healing pathway mediators, were analyzed using STRING database, version 10.5 (http://string-db.org), and evaluated using Cytoscape Software version: Cytoscape_v3.7.1 (https://www.cytoscape.org). Interactions were predicted with a high confidence level of 0.400.

**Figure 7:**
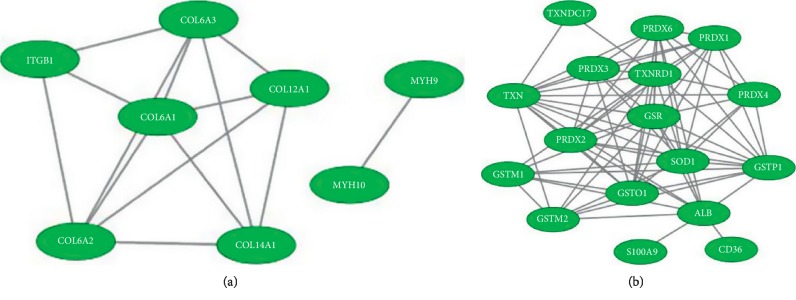
STRING analysis of inflammatory-mediated pathways and cellular detoxification pathways. To determine the protein-protein interactions of the (a) 9 proteins among a total of 252 proteins from the proteome profiling of MFSCC that are inflammatory mediators and (b) 17 proteins among a total of 252 proteins that are cellular detoxification mediators were analyzed using STRING database, version 10.5 (http://string-db.org), and evaluated using Cytoscape Software version: Cytoscape_v3.7.1 (https://www.cytoscape.org). Interactions were predicted with a high confidence level of 0.400.

The gene expression profile of the expressed proteins was obtained in terms of biological process, cellular component, and molecular function that were depicted using web-based gene ontology tool (http://www.webgestalt.org). GO revealed the metabolic process that has taken the major portion of the protein set that is 189 proteins involved in the biological process of these 252 proteins (Figure 3). A total of 198 proteins are involved in protein binding from the molecular function categories. We uploaded the obtained 252 proteins onto the PANTHER database for pathway enrichment analysis, which identified 46 significant signaling pathways. Among the 46 pathways, 19 proteins were involved in the integrin signaling pathway (P00034), and 9 proteins were involved in inflammation mediated by chemokine and cytokine signaling pathway (P00031) is the major pathway enrichments from the identified pathway enrichments (Figure 4). To predict protein-protein interactions and protein complexes, along with the putative pathways, the above proteins were subjected to STRING analysis. STRING generated the interconnected protein network and developed several modules after clustering with a high confidence level of 0.400 (Figure 5). Four putative pathways were selected by STRING analysis for further individual interactions and were evaluated using Cytoscape Software version: Cytoscape_v3.7.1 (https://www.cytoscape.org) (Supplementary Tables [Supplementary-material supplementary-material-1]–[Supplementary-material supplementary-material-1]). Among the 252 proteins, 19 proteins are involved in integrin signaling pathways (Figure 6(a)), 36 proteins are involved in response to wound healing (Figure 6(b)), 9 proteins are involved in inflammatory-mediated pathways (Figure 7(a)), and 17 proteins are involved in cellular detoxification (Figure 7(b)).

### 3.3. Effect of MFSCC on Macrophage RAW264.7 Cell Viability

**Figure 8:**
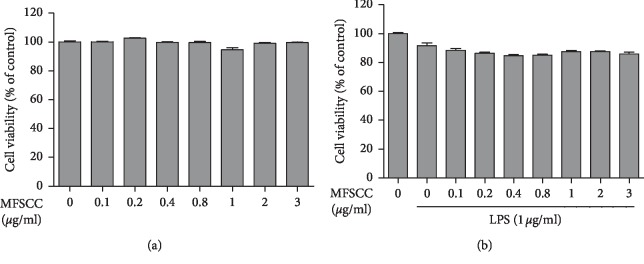
Effect of MFSCC on RAW264.7 cell viability. RAW264.7 macrophage cells were pretreated for 1 h with LPS (1 *μ*g/mL) followed by the treatment with MFSCC at the indicated concentration (0.1 to 3 *μ*g/mL) for 24 h. The viability of RAW264.7 cells in the (a) absence or (b) presence of LPS was assayed using 3-(4,5-dimethylthiazol-2-yl)-2,5-diphenyltetrazolium bromide (MTT).

To find the nontoxic dose of MFSCC in order to measure its anti-inflammatory effect in RAW264.7 cells, we treated with different concentrations of MFSCC (0.1 to 3 *μ*g/mL) alone or pretreated with LPS (1 *μ*g/mL); cells were evaluated by an MTT-based viability assay. After incubating the cells for 24 h (Figures 8(a) and 8(b)), the cell viability was not compromised by the concentration of MFSCC ranging from 0.1 to 3 *μ*g/mL in both LPS pretreated and untreated groups of cells. We also examined the cytotoxicity of MFSCC using the LDH assay (Supplementary [Supplementary-material supplementary-material-1]). The results obtained by the LDH assay revealed that there was no significant cytotoxicity in RAW264.7 cells treated with MFSCC concentration ranging from 0.1 to 3 *μ*g/mL, when compared to positive controls (high control). Therefore, concentrations of MFSCC were made from 0.1 to 2 *μ*g/mL for the study of their anti-inflammatory effects.

### 3.4. MFSCC Inhibits LPS-Induced NO Production

**Figure 9:**
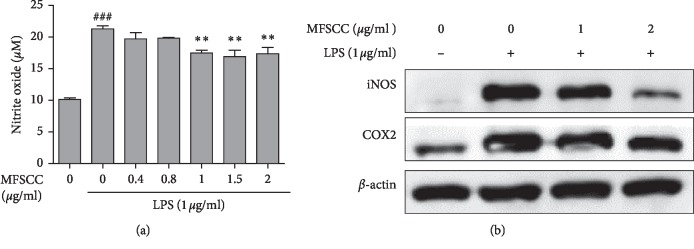
Effect of MFSCC on LPS-induced production NO and proinflammatory mediators in RAW 264.7 cells. (a) NO production was measured in RAW264.7 cells treated with MFSCC at concentrations of 0, 0.4, 0.8, 1, 1.5, and 2 μg/mL for 24h with LPS (1 μg/mL) pretreatment for 1h. (b) Effect of MFSCC on LPS-induced iNOS and COX-2 protein expression levels in RAW264.7 cells. Cells were pretreated with LPS (1μg/mL) for 1h and then treated with MFSCC at concentrations of 0, 1, and 2 μg/mL for 24 h. β‐actin was used as a loading control. The values are expressed as the mean ± SD of three independent experiments (###p < 0.05 vs. untreated; ∗∗p < 0.05 vs. LPS treated).

The effect of MFSCC on NO production in LPS-induced RAW264.7 cells was investigated by the Griess assay. The cells were pretreated with LPS (1 *μ*g/mL), followed by treatment with MFSCC at the indicated concentrations of MFSCC (0, 0.4, 0.8, 1, 1.5, and 2 *μ*g/mL), and incubated for 24 h. As shown in Figure 9(a), treatment with LPS induced a large amount of NO production, which was suppressed upon further treatment with MFSCC in a dose-dependent manner. Thus, compared with the LPS-only treated group, there is a marked decrease in the NO accumulation in the cells cotreated with MFSCC.

### 3.5. MFSCC Inhibits LPS-Induced Protein Expression of COX-2 and iNOS

The effects of MFSCC on COX-2 and iNOS protein expression levels in LPS-induced RAW264.7 cells were evaluated by western blotting. RAW264.7 cells that were stimulated with LPS showed elevated expression of COX-2 and iNOS at protein expression levels when compared with that of LPS-untreated control cells, whereas after treatment with MFSCC, protein expression levels were decreased significantly in RAW264.7 (Figure 9(b)). These results indicate that MFSCC efficiently suppressed LPS-induced COX-2 and iNOS at the protein expression level.

## 4. Discussion

Proteomic analysis is frequently employed in recent times for the identification of protein expression patterns in stem cells to understand their potential to cure several chronic diseases [14]. Investigations of ADSCs have been undertaken for their presumptive use in regenerative medicine based on their antiapoptotic, protrophic, immunomodulatory, and proangiogenic properties [2, 15, 16]. Specifically, ADSCs are significant for the treatments of chronic wounds, as they may reinstate the healing process by controlling inflammation, promoting ingrowth of new vessels into the hypoxic tissue and maintaining migration, fibroblast, and keratinocyte proliferation [16–18]. Recent studies have shown that therapies based on stem cell secretome may present substantial dominance over stem cell-based applications regarding their manufacture, storage without toxic cryopreservation agents, and loss of potency after long periods of culture, immunogenicity, potential infections, and cost- and time-effectiveness. These secretome-containing secreted factors are in the form of cytokines, extracellular vesicles, and growth factors. Thus, the noncell-based agents have a broad range of applications, such as immunomodulatory, antiapoptotic, protrophic, and proangiogenic properties [16, 19]. In this context, the current study data show a preparation of MFSCC and its proteome profile to elucidate the molecular composition and beneficial mechanisms of actions regulated by the identified proteins.

Herein, we explored the complete protein profiling of MFSCC. The Nano-LC-MS/MS analysis data of MFSCC could furnish clues for considering them as noncell stem cell therapeutics for their immunomodulatory and wound healing response, which also enlightens the understanding of its mechanisms at the molecular level. Pathway enrichment analysis and STRING analyses have shown that MFSCC proteins are involved mainly in the integrin pathway, inflammatory response, response to wound healing, and cellular detoxification (Supplementary Tables [Supplementary-material supplementary-material-1]–[Supplementary-material supplementary-material-1]). Among the 252 proteins, 19 proteins are involved in integrin signaling pathways, 36 proteins are involved in response to wound healing, 9 proteins are involved in inflammatory-mediated pathways, and 17 proteins are involved in cellular detoxification. Integrins are the major protein set present in the current protein data from the protein profiling of MFSCC. Integrins play a crucial role in cell-cell adhesion to the extracellular matrix and to different cells, helping ligand binding that stimulated several intracellular pathways, and the effect will be optimal depending upon the cell. Integrin activation has been conjugated to proliferation, secretion of matrix-degrading enzymes, migration, invasion, and cytokine production [20]. In malignant disease, integrin expression was often found to be deregulated and tumors use integrins to evade apoptosis or metastasize, revealing that integrin signaling has to be tightly regulated [21, 22]. The synovial tissue is infiltrated by immune cells that secrete large amounts of cytokines during the course of rheumatoid arthritis, and these proinflammatory milieu's lead to an elevation of integrin receptors and their ligands in the synovial tissue. As a result, integrin signaling is enhanced, leading to the intensified production of cytokines and matrix-degrading enzymes. Integrin alpha-5, integrin alpha-V, integrin beta-1, and integrin beta-3 are the four integrins that are present among the total proteins from MFSCC. The inflammatory response, once it has achieved its proimmunogenic functions and protective nature, becomes a decisive determinant of what might be premeditated as the enigma of inflammation. On the one hand, inflammation is crucial to resolve tissue injury and preserve homeostasis, and while on the other hand, inflammation is a pivotal contributor in the vast majority of human diseases [23]. The present data of protein from MFSCC included some interesting inflammation regulator proteins, wound healing proteins, and integrin signaling pathways proteins, and among them, some are proven to exhibit the anti-inflammatory effect (Supplementary Tables [Supplementary-material supplementary-material-1]–[Supplementary-material supplementary-material-1]). Annexin A1 (ANXA1) is one such protein that has been suggested to be a mediator of the anti-inflammatory actions of glucocorticoids and more recently shown to be an endogenous neuroprotective agent. Annexin A1 protein and its mimetic peptide Ac2-26 displayed anti-inflammatory mechanisms by inhibiting the release of inflammatory mediators independently of the NF-kB-signaling pathway in models of ocular [24]. The involvement of MFSCC protein profile composed of ANXA1 and GO in its wound healing pathway has been shown. Regulatory and biological functions, like antitumor, antimicrobial, antiproliferative, and antinociceptive activities, are mediated by extracellular S100A8, S100A9, and S100A8/A9 proteins. S100A8 (MRP8) and S100A9 (MRP14) are calcium-binding proteins that act as accomplices to the group of damage-associated molecular patterns (DAMPs) and are particularly disclosed in phagocytes, i.e., granulocytes, monocytes, and activated macrophages. Both proteins are expressed uniformly and form a substantial heterodimer S100A8/A9, which ubiquitously appears and is able to stimulate macrophages via the binding and activation of Toll-like receptor (TLR) 4-dependent signaling cascades [25]. Matrix metalloproteinase (MMP) expression in murine and human chondrocytes was directly stimulated by S100A8 and S100A9, thereby promoting the breakdown of cartilage in osteoarthritis and rheumatoid arthritis [26]. In the current study, S100A8 and S100A9 are found to be involved in the wound healing process along with gelsolin (GSN) (Supplementary [Supplementary-material supplementary-material-1]). During acute injury and inflammation levels of plasma, GSN protein levels were decreased, and recombinant plasma GSN administration to animals improves outcomes following burn or sepsis injuries. Animals subjected to systemic inflammation can prolong survival and prevent complications of acute injury by the administration of pGSN [27, 28]. Meanwhile, the cumulative data of MFSCC proteome strongly support the concept of noncell-based stem cell therapeutics to overcome the difficulties of cell-based therapies in various diseases including tissue regeneration, chronic inflammatory diseases, and wound repair.

An inducible iNOS is involved in the immune response-like secretion of proinflammatory cytokines through the induction of high production of NO. NO radical plays a crucial role in regulating inflammation and immune responses in rheumatoid arthritis (RA), inflammatory bowel diseases, and asthma [29]. Scavenging endogenous NO in inflammation condition may be useful for treating chronic inflammatory disorders, including rheumatoid arthritis [30]. As expected, treatment with MFSCC inhibited the NO production in RAW246.7 in a dose-dependent manner, indicating the ability of MFSCC to suppress the production of NO in an inflammatory condition. iNOS and COX-2 have been shown to play pivotal roles in the development of certain chronic inflammatory diseases, and targeting the inhibition of iNOS and COX-2 using anti-inflammatory agents has shown promising outcomes [31]. In general, the protein expression of COX-2 was known to parallel that of iNOS. The protein expression of both iNOS and COX-2 was decreased by MFSCC treatment in LPS-stimulated RAW246.7 cells. The administration of MFSCC has shown anti-inflammatory effect in LPS-stimulated RAW246.7 cells by reducing NO production and also inhibiting iNOS and COX-2. This discordant result may be attributed to the degree of reliance on iNOS and COX-2 promoter's diverse transcription factors.

## 5. Conclusions

Our current results give a booster to the noncell-based stem cell therapeutics research towards using them more strongly for the treatment of regenerative medicine especially towards arthritis and wound healing. Overall, the present findings of the expressed proteins and anti-inflammatory results will lead to a better understating of the potential of noncell-based stem cell therapeutics and will promote the future clinical development of MFSCC for the treatment of chronic inflammatory diseases. Furthermore, disease specific approaches will immensely support for the consideration of MFSCC as cell-free and nontoxic-based stem cell therapeutics for treating chronic disease.

## Data Availability

The authors have included the necessary data supporting their claims for publication. Additional data will be made available on request to the corresponding author.

## Supplementary Materials

Supplementary Materials Table S1: list of total proteins from membrane-free stem cell component (MFSCC) using Nano-LS/MS analysis. Table S2: list of proteins from MFSCC that are involved in the integrin signaling pathway. Table S3: list of proteins from MFSCC that are involved in the inflammatory-mediated pathway. Table S4: list of proteins from MFSCC that are involved in the wound healing pathway. Table S5: list of proteins from MFSCC that are involved in the cellular detoxification pathway. Figure S1: cytotoxic effect of MFSCC on RAW264.7 by LDH assay. RAW264.7 macrophage cells were treated with MFSCC at the indicated concentration of 0.1 to *3 μ*g/mL for 24 h. Cell cytotoxicity assay kit (Duzen Bio Co., Ltd., Guro-gu, Republic of Korea) was used, according to the manufacturer's instructions.

## References

[1] Watt F. M., Driskell R. R. (2010). The therapeutic potential of stem cells. *Philosophical Transactions of the Royal Society of London. Series B, Biological Sciences*.

[2] Pak J., Lee J. H., Lee S. H. (2014). Regenerative repair of damaged meniscus with autologous adipose tissue-derived stem cells. *BioMed Research International*.

[3] Bruun K., Schermer E., Sivendra A. (2018). Therapeutic applications of adipose-derived stem cells in cardiovascular disease. *American Journal of Stem Cells*.

[4] Rybalko V., Hsieh P. L., Ricles L. M., Chung E., Farrar R. P., Suggs L. J. (2017). Therapeutic potential of adipose-derived stem cells and macrophages for ischemic skeletal muscle repair. *Regenerative Medicine*.

[5] Park M. J., Kwok S. K., Lee S. H., Kim E. K., Park S. H., Cho M. L. (2015). Adipose tissue-derived mesenchymal stem cells induce expansion of interleukin-10-producing regulatory B cells and ameliorate autoimmunity in a murine model of systemic lupus erythematosus. *Cell Transplantation*.

[6] Lim M. H., Ong W. K., Sugii S. (2014). The current landscape of adipose-derived stem cells in clinical applications. *Expert Reviews in Molecular Medicine*.

[7] Hong S. J., Traktuev D. O., March K. L. (2010). Therapeutic potential of adipose-derived stem cells in vascular growth and tissue repair. *Current Opinion in Organ Transplantation*.

[8] Platas J., Guillen M. I., del Caz M. D. P., Gomar F., Mirabet V., Alcaraz M. J. (2013). Conditioned media from adipose-tissue-derived mesenchymal stem cells downregulate degradative mediators induced by interleukin-1beta in osteoarthritic chondrocytes. *Mediators of Inflammation*.

[9] Miller R. A., Spellman D. S. (2014). Mass spectrometry-based biomarkers in drug development. *Advancements of Mass Spectrometry in Biomedical Research*.

[10] Schirle M., Bantscheff M., Kuster B. (2012). Mass spectrometry-based proteomics in preclinical drug discovery. *Chemistry & Biology*.

[11] Tuli L., Ressom H. W. (2009). LC-MS based detection of differential protein expression. *Journal of Proteomics & Bioinformatics*.

[12] Novak A., Amit M., Ziv T. (2012). Proteomics profiling of human embryonic stem cells in the early differentiation stage. *Stem Cell Reviews and Reports*.

[13] Gaspari M., Cuda G. (2011). Nano LC-MS/MS: a robust setup for proteomic analysis. *Methods in Molecular Biology*.

[14] Lee S. K., Kim J. H., Kim S. S. (2013). Profiling and semiquantitative analysis of the cell surface proteome in human mesenchymal stem cells. *Analytical and Bioanalytical Chemistry*.

[15] Falomo M. E., Ferroni L., Tocco I., Gardin C., Zavan B. (2015). Immunomodulatory role of adipose-derived stem cells on equine endometriosis. *BioMed Research International*.

[16] Kapur S. K., Katz A. J. (2013). Review of the adipose derived stem cell secretome. *Biochimie*.

[17] Brayfield C., Marra K., Rubin J. P. (2010). Adipose stem cells for soft tissue regeneration. *Handchirurgie, Mikrochirurgie, plastische Chirurgie*.

[18] Hassan W. U., Greiser U., Wang W. (2014). Role of adipose-derived stem cells in wound healing. *Wound Repair and Regeneration*.

[19] Blazquez R., Sanchez-Margallo F. M., de la Rosa O. (2014). Immunomodulatory potential of human adipose mesenchymal stem cells derived exosomes on in vitro stimulated T cells. *Frontiers in Immunology*.

[20] Lowin T., Straub R. H. (2011). Integrins and their ligands in rheumatoid arthritis. *Arthritis Research & Therapy*.

[21] Ganguly K. K., Pal S., Moulik S., Chatterjee A. (2013). Integrins and metastasis. *Cell Adhesion & Migration*.

[22] Desgrosellier J. S., Cheresh D. A. (2010). Integrins in cancer: biological implications and therapeutic opportunities. *Nature Reviews Cancer*.

[23] Gallo J., Raska M., Kriegova E., Goodman S. B. (2017). Inflammation and its resolution and the musculoskeletal system. *Journal of Orthopaedic Translation*.

[24] Girol A. P., Mimura K. K. O., Drewes C. C. (2013). Anti-inflammatory mechanisms of the annexin A1 protein and its mimetic peptide Ac2-26 in models of ocular inflammation in vivo and in vitro. *The Journal of Immunology*.

[25] Srikrishna G. (2012). S100A8 and S100A9: new insights into their roles in malignancy. *Journal of Innate Immunity*.

[26] Ryckman C., Vandal K., Rouleau P., Talbot M., Tessier P. A. (2003). Proinflammatory activities of S100: proteins S100A8, S100A9, and S100A8/A9 induce neutrophil chemotaxis and adhesion. *The Journal of Immunology*.

[27] Osborn T. M., Verdrengh M., Stossel T. P., Tarkowski A., Bokarewa M. (2008). Decreased levels of the gelsolin plasma isoform in patients with rheumatoid arthritis. *Arthritis Research & Therapy*.

[28] Li G. H., Arora P. D., Chen Y., McCulloch C. A., Liu P. (2012). Multifunctional roles of gelsolin in health and diseases. *Medicinal Research Reviews*.

[29] Chen L., Deng H., Cui H. (2018). Inflammatory responses and inflammation-associated diseases in organs. *Oncotarget*.

[30] Arulselvan P., Fard M. T., Tan W. S. (2016). Role of antioxidants and natural products in inflammation. *Oxidative Medicine and Cellular Longevity*.

[31] Moita E., Gil-Izquierdo A., Sousa C. (2013). Integrated analysis of COX-2 and iNOS derived inflammatory mediators in LPS-stimulated RAW macrophages pre-exposed to Echium plantagineum L. bee pollen extract. *PLoS One*.

