# Supplementary material for: Proteome Profiling of Membrane-Free Stem Cell Components by Nano-LS/MS Analysis and Its Anti-Inflammatory Activity
Source: Evid Based Complement Alternat Med. 2019 Nov 4;2019:4683272. doi: 10.1155/2019/4683272 (PMC6875034; doi:10.1155/2019/4683272)

**Table-S1**

| **S.No** | **Identified Proteins (379)** | **Accession No** | **Uniprot ID** | **Molecular Weight** | **Peptides Matched** |
| --- | --- | --- | --- | --- | --- |
| 1 | Vimentin | VIME_HUMAN | P08670 | 54 kDa | 252 |
| 2 | Keratin, type II cytoskeletal 1 | K2C1_HUMAN | P04264 | 66 kDa | 142 |
| 3 | Filamin-A | FLNA_HUMAN | P21333 | 281 kDa | 130 |
| 4 | Annexin A2 | ANXA2_HUMAN | P07355 | 39 kDa | 127 |
| 5 | Keratin, type I cytoskeletal 10 | K1C10_HUMAN | P13645 | 59 kDa | 82 |
| 6 | Keratin, type I cytoskeletal 9 | K1C9_HUMAN | P35527 | 62 kDa | 82 |
| 7 | Prelamin-A/C | LMNA_HUMAN | P02545 | 74 kDa | 63 |
| 8 | Myosin-9 | MYH9_HUMAN | P35579 | 227 kDa | 62 |
| 9 | Keratin, type II cytoskeletal 2 | K22E_HUMAN | P35908 | 65 kDa | 59 |
| 10 | Glyceraldehyde-3-phosphate dehydrogenase | G3P_HUMAN | P04406 | 36 kDa | 57 |
| 11 | Annexin A5 | ANXA5_HUMAN | P08758 | 36 kDa | 56 |
| 12 | Aminopeptidase N | AMPN_HUMAN | P15144 | 110 kDa | 56 |
| 13 | Annexin A1 | ANXA1_HUMAN | P04083 | 39 kDa | 49 |
| 14 | Collagen alpha-3(VI) chain | CO6A3_HUMAN | P12111 | 344 kDa | 48 |
| 15 | Neprilysin | NEP_HUMAN | P08473 | 86 kDa | 48 |
| 16 | Early endosome antigen 1 | EEA1_HUMAN | Q15075 | 162 kDa | 46 |
| 17 | Alpha-actinin-1 | ACTN1_HUMAN | P12814 | 103 kDa | 45 |
| 18 | Filamin-C | FLNC_HUMAN | Q14315 | 291 kDa | 44 |
| 19 | Cathepsin D | CATD_HUMAN | P07339 | 45 kDa | 44 |
| 20 | Myosin-10 | MYH10_HUMAN | P35580 | 229 kDa | 44 |
| 21 | Keratin, type I cytoskeletal 16 | K1C16_HUMAN | P08779 | 51 kDa | 42 |
| 22 | Filamin-B | FLNB_HUMAN | O75369 | 278 kDa | 37 |
| 23 | Alpha-enolase | ENOA_HUMAN | P06733 | 47 kDa | 36 |
| 24 | Myoferlin | MYOF_HUMAN | Q9NZM1 | 235 kDa | 36 |
| 25 | Keratin, type II cytoskeletal 6C | K2C6C_HUMAN | P48668 | 60 kDa | 35 |
| 26 | Lysosome-associated membrane glycoprotein 1 | LAMP1_HUMAN | P11279 | 45 kDa | 34 |
| 27 | Vinculin | VINC_HUMAN | P18206 | 124 kDa | 34 |
| 28 | Voltage-dependent anion-selective channel protein 1 | VDAC1_HUMAN | P21796 | 31 kDa | 34 |
| 29 | Glycine--tRNA ligase | SYG_HUMAN | P41250 | 83 kDa | 32 |
| 30 | Erythrocyte band 7 integral membrane protein | STOM_HUMAN | P27105 | 32 kDa | 31 |
| 31 | Lysosome membrane protein 2 | SCRB2_HUMAN | Q14108 | 54 kDa | 29 |
| 32 | Dipeptidyl peptidase 4 | DPP4_HUMAN | P27487 | 88 kDa | 28 |
| 33 | Keratin, type II cytoskeletal 5 | K2C5_HUMAN | P13647 | 62 kDa | 25 |
| 34 | Transmembrane glycoprotein NMB | GPNMB_HUMAN | Q14956 | 64 kDa | 24 |
| 35 | Ras GTPase-activating-like protein IQGAP1 | IQGA1_HUMAN | P46940 | 189 kDa | 23 |
| 36 | Cathepsin K | CATK_HUMAN | P43235 | 37 kDa | 23 |
| 37 | Prolyl endopeptidase FAP | SEPR_HUMAN | Q12884 | 88 kDa | 22 |
| 38 | Annexin A4 | ANXA4_HUMAN | P09525 | 36 kDa | 22 |
| 39 | Glucosylceramidase | GLCM_HUMAN | P04062 | 60 kDa | 22 |
| 40 | Fructose-bisphosphate aldolase A | ALDOA_HUMAN | P04075 | 39 kDa | 22 |
| 41 | Protein disulfide-isomerase A4 | PDIA4_HUMAN | P13667 | 73 kDa | 21 |
| 42 | Glutathione S-transferase P | GSTP1_HUMAN | P09211 | 23 kDa | 21 |
| 43 | Serpin B6 | SPB6_HUMAN | P35237 | 43 kDa | 21 |
| 44 | Peroxiredoxin-4 | PRDX4_HUMAN | Q13162 | 31 kDa | 21 |
| 45 | Protein disulfide-isomerase | PDIA1_HUMAN | P07237 | 57 kDa | 20 |
| 46 | Nucleoside diphosphate kinase B | NDKB_HUMAN | P22392 | 17 kDa | 20 |
| 47 | Keratin, type I cytoskeletal 14 | K1C14_HUMAN | P02533 | 52 kDa | 19 |
| 48 | Thioredoxin-dependent peroxide reductase, mitochondrial | PRDX3_HUMAN | P30048 | 28 kDa | 19 |
| 49 | Plectin | PLEC_HUMAN | Q15149 | 532 kDa | 19 |
| 50 | Alpha-actinin-4 | ACTN4_HUMAN | O43707 | 105 kDa | 19 |
| 51 | Superoxide dismutase [Cu-Zn] | SODC_HUMAN | P00441 | 16 kDa | 19 |
| 52 | Nucleolin | NUCL_HUMAN | P19338 | 77 kDa | 18 |
| 53 | 60S ribosomal protein L6 | RL6_HUMAN | Q02878 | 33 kDa | 18 |
| 54 | Integrin alpha-V | ITAV_HUMAN | P06756 | 116 kDa | 17 |
| 55 | Annexin A6 | ANXA6_HUMAN | P08133 | 76 kDa | 17 |
| 56 | Cathepsin B | CATB_HUMAN | P07858 | 38 kDa | 17 |
| 57 | ATP synthase subunit alpha, mitochondrial | ATPA_HUMAN | P25705 | 60 kDa | 16 |
| 58 | Keratin, type I cytoskeletal 13 | K1C13_HUMAN | P13646 | 50 kDa | 16 |
| 59 | Syntenin-1 | SDCB1_HUMAN | O00560 | 32 kDa | 16 |
| 60 | Thioredoxin reductase 1, cytoplasmic | TRXR1_HUMAN | Q16881 | 71 kDa | 16 |
| 61 | Prolow-density lipoprotein receptor-related protein 1 | LRP1_HUMAN | Q07954 | 505 kDa | 15 |
| 62 | Peroxiredoxin-2 | PRDX2_HUMAN | P32119 | 22 kDa | 15 |
| 63 | Ferritin heavy chain | FRIH_HUMAN | P02794 | 21 kDa | 15 |
| 64 | Keratin, type II cytoskeletal 4 | K2C4_HUMAN | P19013 | 57 kDa | 15 |
| 65 | L-lactate dehydrogenase A chain | LDHA_HUMAN | P00338 | 37 kDa | 15 |
| 66 | Collagen alpha-1(XII) chain | COCA1_HUMAN | Q99715 | 333 kDa | 15 |
| 67 | 60 kDa heat shock protein, mitochondrial | CH60_HUMAN | P10809 | 61 kDa | 14 |
| 68 | Prenylcysteine oxidase 1 | PCYOX_HUMAN | Q9UHG3 | 57 kDa | 14 |
| 69 | Histone H3.1t | H31T_HUMAN | Q16695 | 16 kDa | 14 |
| 70 | N-acetylglucosamine-6-sulfatase | GNS_HUMAN | P15586 | 62 kDa | 14 |
| 71 | Prohibitin-2 | PHB2_HUMAN | Q99623 | 33 kDa | 14 |
| 72 | Matrix metalloproteinase-14 | MMP14_HUMAN | P50281 | 66 kDa | 14 |
| 73 | Glucose-6-phosphate 1-dehydrogenase | G6PD_HUMAN | P11413 | 59 kDa | 14 |
| 74 | Lysosome-associated membrane glycoprotein 2 | LAMP2_HUMAN | P13473 | 45 kDa | 13 |
| 75 | Lysosomal alpha-glucosidase | LYAG_HUMAN | P10253 | 105 kDa | 13 |
| 76 | 60S ribosomal protein L7 | RL7_HUMAN | P18124 | 29 kDa | 12 |
| 77 | Collagen alpha-1(I) chain | CO1A1_HUMAN | P02452 | 139 kDa | 12 |
| 78 | Beta-hexosaminidase subunit beta | HEXB_HUMAN | P07686 | 63 kDa | 12 |
| 79 | ATP synthase subunit beta, mitochondrial | ATPB_HUMAN | P06576 | 57 kDa | 12 |
| 80 | Ferritin light chain | FRIL_HUMAN | P02792 | 20 kDa | 12 |
| 81 | Pyruvate kinase PKM | KPYM_HUMAN | P14618 | 58 kDa | 11 |
| 82 | Neuroblast differentiation-associated protein AHNAK | AHNK_HUMAN | Q09666 | 629 kDa | 11 |
| 83 | Unconventional myosin-Id | MYO1D_HUMAN | O94832 | 116 kDa | 11 |
| 84 | Fatty acid-binding protein, heart | FABPH_HUMAN | P05413 | 15 kDa | 11 |
| 85 | Peroxiredoxin-1 | PRDX1_HUMAN | Q06830 | 22 kDa | 11 |
| 86 | Serpin H1 | SERPH_HUMAN | P50454 | 46 kDa | 11 |
| 87 | Annexin A7 | ANXA7_HUMAN | P20073 | 53 kDa | 11 |
| 88 | Hornerin | HORN_HUMAN | Q86YZ3 | 282 kDa | 11 |
| 89 | UDP-glucose 6-dehydrogenase | UGDH_HUMAN | O60701 | 55 kDa | 11 |
| 90 | Fibronectin | FINC_HUMAN | P02751 | 263 kDa | 11 |
| 91 | Dihydrolipoyllysine-residue succinyltransferase component of 2-oxoglutarate dehydrogenase complex, mitochondrial | ODO2_HUMAN | P36957 | 49 kDa | 11 |
| 92 | CD44 antigen | CD44_HUMAN | P16070 | 82 kDa | 11 |
| 93 | Glutathione S-transferase omega-1 | GSTO1_HUMAN | P78417 | 28 kDa | 11 |
| 94 | CD59 glycoprotein | CD59_HUMAN | P13987 | 14 kDa | 10 |
| 95 | Protein disulfide-isomerase A6 | PDIA6_HUMAN | Q15084 | 48 kDa | 10 |
| 96 | Integrin beta-1 | ITB1_HUMAN | P05556 | 88 kDa | 10 |
| 97 | Galectin-3-binding protein | LG3BP_HUMAN | Q08380 | 65 kDa | 10 |
| 98 | Transmembrane protein 43 | TMM43_HUMAN | Q9BTV4 | 45 kDa | 10 |
| 99 | Nicastrin | NICA_HUMAN | Q92542 | 78 kDa | 10 |
| 100 | Lysosomal Pro-X carboxypeptidase | PCP_HUMAN | P42785 | 56 kDa | 10 |
| 101 | Platelet glycoprotein 4 | CD36_HUMAN | P16671 | 53 kDa | 10 |
| 102 | Transmembrane emp24 domain-containing protein 10 | TMEDA_HUMAN | P49755 | 25 kDa | 10 |
| 103 | Gelsolin | GELS_HUMAN | P06396 | 86 kDa | 10 |
| 104 | Leukocyte elastase inhibitor | ILEU_HUMAN | P30740 | 43 kDa | 10 |
| 105 | Lamin-B2 | LMNB2_HUMAN | Q03252 | 70 kDa | 10 |
| 106 | Transaldolase | TALDO_HUMAN | P37837 | 38 kDa | 9 |
| 107 | Galectin-3 | LEG3_HUMAN | P17931 | 26 kDa | 9 |
| 108 | Chloride intracellular channel protein 1 | CLIC1_HUMAN | O00299 | 27 kDa | 9 |
| 109 | Elongation factor 1-gamma | EF1G_HUMAN | P26641 | 50 kDa | 9 |
| 110 | Glutathione synthetase | GSHB_HUMAN | P48637 | 52 kDa | 9 |
| 111 | Annexin A11 | ANX11_HUMAN | P50995 | 54 kDa | 9 |
| 112 | Collagen alpha-2(I) chain | CO1A2_HUMAN | P08123 | 129 kDa | 9 |
| 113 | Thioredoxin | THIO_HUMAN | P10599 | 12 kDa | 9 |
| 114 | Synaptic vesicle membrane protein VAT-1 homolog | VAT1_HUMAN | Q99536 | 42 kDa | 9 |
| 115 | Alpha-aminoadipic semialdehyde dehydrogenase | AL7A1_HUMAN | P49419 | 58 kDa | 9 |
| 116 | Acid ceramidase | ASAH1_HUMAN | Q13510 | 45 kDa | 9 |
| 117 | Transketolase | TKT_HUMAN | P29401 | 68 kDa | 9 |
| 118 | Talin-1 | TLN1_HUMAN | Q9Y490 | 270 kDa | 9 |
| 119 | Putative phospholipase B-like 2 | PLBL2_HUMAN | Q8NHP8 | 65 kDa | 9 |
| 120 | ADP-ribosyl cyclase/cyclic ADP-ribose hydrolase 2 | BST1_HUMAN | Q10588 | 36 kDa | 8 |
| 121 | Farnesyl pyrophosphate synthase | FPPS_HUMAN | P14324 | 48 kDa | 8 |
| 122 | Heterogeneous nuclear ribonucleoprotein Q | HNRPQ_HUMAN | O60506 | 70 kDa | 8 |
| 123 | 60S ribosomal protein L14 | RL14_HUMAN | P50914 | 23 kDa | 8 |
| 124 | Beta-galactosidase | BGAL_HUMAN | P16278 | 76 kDa | 8 |
| 125 | Glia-derived nexin | GDN_HUMAN | P07093 | 44 kDa | 8 |
| 126 | 60S ribosomal protein L5 | RL5_HUMAN | P46777 | 34 kDa | 8 |
| 127 | Serum albumin | ALBU_HUMAN | P02768 | 69 kDa | 8 |
| 128 | Collagen alpha-1(VI) chain | CO6A1_HUMAN | P12109 | 109 kDa | 8 |
| 129 | Angiotensin-converting enzyme | ACE_HUMAN | P12821 | 150 kDa | 8 |
| 130 | Calnexin | CALX_HUMAN | P27824 | 68 kDa | 8 |
| 131 | Malate dehydrogenase, mitochondrial | MDHM_HUMAN | P40926 | 36 kDa | 8 |
| 132 | Niemann-Pick C1 protein | NPC1_HUMAN | O15118 | 142 kDa | 8 |
| 133 | Tropomyosin beta chain | TPM2_HUMAN | P07951 | 33 kDa | 8 |
| 134 | Procollagen-lysine,2-oxoglutarate 5-dioxygenase 1 | PLOD1_HUMAN | Q02809 | 84 kDa | 8 |
| 135 | Spectrin alpha chain, non-erythrocytic 1 | SPTN1_HUMAN | Q13813 | 285 kDa | 8 |
| 136 | Single-stranded DNA-binding protein, mitochondrial | SSBP_HUMAN | Q04837 | 17 kDa | 7 |
| 137 | Trifunctional enzyme subunit alpha, mitochondrial | ECHA_HUMAN | P40939 | 83 kDa | 7 |
| 138 | ADP-sugar pyrophosphatase | NUDT5_HUMAN | Q9UKK9 | 24 kDa | 7 |
| 139 | 60S acidic ribosomal protein P0-like | RLA0L_HUMAN | Q8NHW5 | 34 kDa | 7 |
| 140 | Tripeptidyl-peptidase 1 | TPP1_HUMAN | O14773 | 61 kDa | 7 |
| 141 | Leukocyte surface antigen CD47 | CD47_HUMAN | Q08722 | 35 kDa | 7 |
| 142 | Cytoskeleton-associated protein 4 | CKAP4_HUMAN | Q07065 | 66 kDa | 7 |
| 143 | Collagen alpha-1(XIV) chain | COEA1_HUMAN | Q05707 | 194 kDa | 7 |
| 144 | Lactadherin | MFGM_HUMAN | Q08431 | 43 kDa | 7 |
| 145 | HLA class I histocompatibility antigen, B-54 alpha chain | 1B54_HUMAN | P30492 | 40 kDa | 7 |
| 146 | Moesin O | MOES_HUMAN | P26038 | 68 kDa | 7 |
| 147 | Collagen triple helix repeat-containing protein 1 | CTHR1_HUMAN | Q96CG8 | 26 kDa | 7 |
| 148 | Glyoxalase domain-containing protein 4 | GLOD4_HUMAN | Q9HC38 | 35 kDa | 7 |
| 149 | Histone H2A type 1-D | H2A1D_HUMAN | P20671 | 14 kDa | 7 |
| 150 | Lysosomal protective protein | PPGB_HUMAN | P10619 | 54 kDa | 7 |
| 151 | Thrombospondin-1 | TSP1_HUMAN | P07996 | 129 kDa | 7 |
| 152 | Collagen alpha-2(VI) chain | CO6A2_HUMAN | P12110 | 109 kDa | 7 |
| 153 | Galectin-1 | LEG1_HUMAN | P09382 | 15 kDa | 7 |
| 154 | Dolichyl-diphosphooligosaccharide--protein glycosyltransferase 48 kDa subunit | OST48_HUMAN | P39656 | 51 kDa | 7 |
| 155 | Polymerase I and transcript release factor | PTRF_HUMAN | Q6NZI2 | 43 kDa | 7 |
| 156 | Peptidyl-prolyl cis-trans isomerase B | PPIB_HUMAN | P23284 | 24 kDa | 6 |
| 157 | CD63 antigen | CD63_HUMAN | P08962 | 26 kDa | 6 |
| 158 | 5'-nucleotidase | 5NTD_HUMAN | P21589 | 63 kDa | 6 |
| 159 | 2',3'-cyclic-nucleotide 3'-phosphodiesterase | CN37_HUMAN | P09543 | 48 kDa | 6 |
| 160 | Lanosterol synthase | ERG7_HUMAN | P48449 | 83 kDa | 6 |
| 161 | Protein S100-A4 | S10A4_HUMAN | P26447 | 12 kDa | 6 |
| 162 | Xaa-Pro aminopeptidase 2 | XPP2_HUMAN | O43895 | 76 kDa | 6 |
| 163 | 60S ribosomal protein L3 | RL3_HUMAN | P39023 | 46 kDa | 6 |
| 164 | N-sulphoglucosamine sulphohydrolase | SPHM_HUMAN | P51688 | 57 kDa | 6 |
| 165 | Peroxisomal multifunctional enzyme type 2 | DHB4_HUMAN | P51659 | 80 kDa | 6 |
| 166 | Acylamino-acid-releasing enzyme | ACPH_HUMAN | P13798 | 81 kDa | 6 |
| 167 | 2,4-dienoyl-CoA reductase, mitochondrial | DECR_HUMAN | Q16698 | 36 kDa | 6 |
| 168 | Fascin | FSCN1_HUMAN | Q16658 | 55 kDa | 6 |
| 169 | Keratin, type I cytoskeletal 17 | K1C17_HUMAN | Q04695 | 48 kDa | 6 |
| 170 | Thioredoxin domain-containing protein 17 | TXD17_HUMAN | Q9BRA2 | 14 kDa | 6 |
| 171 | Plexin-B2 | PLXB2_HUMAN | O15031 | 205 kDa | 6 |
| 172 | Voltage-dependent anion-selective channel protein 3 | VDAC3_HUMAN | Q9Y277 | 31 kDa | 6 |
| 173 | Myosin regulatory light chain 12A | ML12A_HUMAN | P19105 | 20 kDa | 6 |
| 174 | Glutathione S-transferase Mu 1 | GSTM1_HUMAN | P09488 | 26 kDa | 5 |
| 175 | Plexin domain-containing protein 2 | PXDC2_HUMAN | Q6UX71 | 60 kDa | 5 |
| 176 | Spectrin beta chain, non-erythrocytic 1 | SPTB2_HUMAN | Q01082 | 275 kDa | 5 |
| 177 | 3-hydroxybutyrate dehydrogenase type 2 | BDH2_HUMAN | Q9BUT1 | 27 kDa | 5 |
| 178 | Protein S100-A9 | S10A9_HUMAN | P06702 | 13 kDa | 5 |
| 179 | DNA-(apurinic or apyrimidinic site) lyase | APEX1_HUMAN | P27695 | 36 kDa | 5 |
| 180 | Platelet-derived growth factor receptor beta | PGFRB_HUMAN | P09619 | 124 kDa | 5 |
| 181 | Vasorin | VASN_HUMAN | Q6EMK4 | 72 kDa | 5 |
| 182 | Dermcidin | DCD_HUMAN | P81605 | 11 kDa | 5 |
| 183 | Adenine phosphoribosyltransferase | APT_HUMAN | P07741 | 20 kDa | 5 |
| 184 | Ribonuclease inhibitor | RINI_HUMAN | P13489 | 50 kDa | 5 |
| 185 | 4F2 cell-surface antigen heavy chain | 4F2_HUMAN | P08195 | 68 kDa | 5 |
| 186 | Dipeptidyl peptidase 2 | DPP2_HUMAN | Q9UHL4 | 54 kDa | 5 |
| 187 | Glutathione reductase, mitochondrial | GSHR_HUMAN | P00390 | 56 kDa | 5 |
| 188 | Calcium/calmodulin-dependent protein kinase type II subunit delta | KCC2D_HUMAN | Q13557 | 56 kDa | 5 |
| 189 | Major vault protein | MVP_HUMAN | Q14764 | 99 kDa | 5 |
| 190 | Epididymal secretory protein E1 | NPC2_HUMAN | P61916 | 17 kDa | 5 |
| 191 | 60S ribosomal protein L28 | RL28_HUMAN | P46779 | 16 kDa | 5 |
| 192 | Phospholipase D3 | PLD3_HUMAN | Q8IV08 | 55 kDa | 5 |
| 193 | UPF0160 protein MYG1, mitochondrial | MYG1_HUMAN | Q9HB07 | 42 kDa | 5 |
| 194 | SH3 domain-binding glutamic acid-rich-like protein | SH3L1_HUMAN | O75368 | 13 kDa | 5 |
| 195 | Glycosylated lysosomal membrane protein | GLMP_HUMAN | Q8WWB7 | 44 kDa | 5 |
| 196 | UDP-glucose:glycoprotein glucosyltransferase 1 | UGGG1_HUMAN | Q9NYU2 | 177 kDa | 5 |
| 197 | Protein S100-A8 | S10A8_HUMAN | P05109 | 11 kDa | 5 |
| 198 | N(G),N(G)-dimethylarginine dimethylaminohydrolase 2 | DDAH2_HUMAN | O95865 | 30 kDa | 5 |
| 199 | N-acetylgalactosamine-6-sulfatase | GALNS_HUMAN | P34059 | 58 kDa | 5 |
| 200 | Integrin beta-3 | ITB3_HUMAN | P05106 | 87 kDa | 5 |
| 201 | Alkaline phosphatase, tissue-nonspecific isozyme | PPBT_HUMAN | P05186 | 57 kDa | 5 |
| 202 | Transmembrane emp24 domain-containing protein 7 | TMED7_HUMAN | Q9Y3B3 | 25 kDa | 5 |
| 203 | Adenosine deaminase | ADA_HUMAN | P00813 | 41 kDa | 5 |
| 204 | Laminin subunit gamma-1 | LAMC1_HUMAN | P11047 | 178 kDa | 5 |
| 205 | Peroxiredoxin-6 | PRDX6_HUMAN | P30041 | 25 kDa | 5 |
| 206 | Tyrosine-protein phosphatase non-receptor type substrate 1 | SHPS1_HUMAN | P78324 | 55 kDa | 5 |
| 207 | CD166 antigen | CD166_HUMAN | Q13740 | 65 kDa | 5 |
| 208 | Cofilin-1 | COF1_HUMAN | P23528 | 19 kDa | 5 |
| 209 | EH domain-containing protein 2 | EHD2_HUMAN | Q9NZN4 | 61 kDa | 5 |
| 210 | 60S ribosomal protein L9 | RL9_HUMAN | P32969 | 22 kDa | 5 |
| 211 | Citrate synthase, mitochondrial | CISY_HUMAN | O75390 | 52 kDa | 5 |
| 212 | Nicotinamide N-methyltransferase | NNMT_HUMAN | P40261 | 30 kDa | 5 |
| 213 | NADH-cytochrome b5 reductase 3 | NB5R3_HUMAN | P00387 | 34 kDa | 5 |
| 214 | Saccharopine dehydrogenase-like oxidoreductase | SCPDL_HUMAN | Q8NBX0 | 47 kDa | 5 |
| 215 | Brain acid soluble protein 1 | BASP1_HUMAN | P80723 | 23 kDa | 5 |
| 216 | Trifunctional enzyme subunit beta, mitochondrial | ECHB_HUMAN | P55084 | 51 kDa | 5 |
| 217 | Intercellular adhesion molecule 1 | ICAM1_HUMAN | P05362 | 58 kDa | 5 |
| 218 | Ribosyldihydronicotinamide dehydrogenase [quinone] | NQO2_HUMAN | P16083 | 26 kDa | 5 |
| 219 | Delta(3,5)-Delta(2,4)-dienoyl-CoA isomerase, mitochondrial | ECH1_HUMAN | Q13011 | 36 kDa | 5 |
| 220 | Heat shock protein beta-1 | HSPB1_HUMAN | P04792 | 23 kDa | 4 |
| 221 | CD151 antigen | CD151_HUMAN | P48509 | 28 kDa | 4 |
| 222 | Cathepsin S | CATS_HUMAN | P25774 | 37 kDa | 4 |
| 223 | Ribosome-binding protein 1 | RRBP1_HUMAN | Q9P2E9 | 152 kDa | 4 |
| 224 | Complement component 1 Q subcomponent-binding protein, mitochondrial | C1QBP_HUMAN | Q07021 | 31 kDa | 4 |
| 225 | Elongation factor Tu, mitochondrial | EFTU_HUMAN | P49411 | 50 kDa | 4 |
| 226 | Integrin alpha-5 | ITA5_HUMAN | P08648 | 115 kDa | 4 |
| 227 | Regulator of nonsense transcripts 1 | RENT1_HUMAN | Q92900 | 124 kDa | 4 |
| 228 | Selenium-binding protein 1 | SBP1_HUMAN | Q13228 | 52 kDa | 4 |
| 229 | Glutathione S-transferase Mu 2 2 | GSTM2_HUMAN | P28161 | 26 kDa | 4 |
| 230 | Heme-binding protein 2 | HEBP2_HUMAN | Q9Y5Z4 | 23 kDa | 4 |
| 231 | Lamin-B1 | LMNB1_HUMAN | P20700 | 66 kDa | 4 |
| 232 | Retinoid-inducible serine carboxypeptidase | RISC_HUMAN | Q9HB40 | 51 kDa | 4 |
| 233 | Sulfide:quinone oxidoreductase, mitochondrial | SQRD_HUMAN | Q9Y6N5 | 50 kDa | 4 |
| 234 | Carboxypeptidase Q | CBPQ_HUMAN | Q9Y646 | 52 kDa | 4 |
| 235 | Dolichyl-diphosphooligosaccharide--protein glycosyltransferase subunit 1 | RPN1_HUMAN | P04843 | 69 kDa | 4 |
| 236 | Ras-related protein Ral-A | RALA_HUMAN | P11233 | 24 kDa | 4 |
| 237 | Sodium/potassium-transporting ATPase subunit beta-3 | AT1B3_HUMAN | P54709 | 32 kDa | 4 |
| 238 | P2X purinoceptor 4 | P2RX4_HUMAN | Q99571 | 43 kDa | 4 |
| 239 | Neutral alpha-glucosidase AB | GANAB_HUMAN | Q14697 | 107 kDa | 4 |
| 240 | Epidermal growth factor receptor | EGFR_HUMAN | P00533 | 134 kDa | 4 |
| 241 | Protein SETSIP | SETLP_HUMAN | P0DME0 | 35 kDa | 4 |
| 242 | Thy-1 membrane glycoprotein | THY1_HUMAN | P04216 | 18 kDa | 4 |
| 243 | YTH domain-containing family protein 1 | YTHD1_HUMAN | Q9BYJ9 | 61 kDa | 4 |
| 244 | Cathepsin Z | CATZ_HUMAN | Q9UBR2 | 34 kDa | 4 |
| 245 | CD9 antigen | CD9_HUMAN | P21926 | 25 kDa | 4 |
| 246 | Alanine--tRNA ligase, cytoplasmic | SYAC_HUMAN | P49588 | 107 kDa | 4 |
| 247 | Programmed cell death 6-interacting protein | PDC6I_HUMAN | Q8WUM4 | 96 kDa | 4 |
| 248 | Aminoacyl tRNA synthase complex-interacting multifunctional protein 1 | AIMP1_HUMAN | Q12904 | 34 kDa | 4 |
| 249 | GDH/6PGL endoplasmic bifunctional protein | G6PE_HUMAN | O95479 | 89 kDa | 4 |
| 250 | Serine protease HTRA1 | HTRA1_HUMAN | Q92743 | 51 kDa | 4 |
| 251 | Keratin, type II cytoskeletal 6B | K2C6B_HUMAN | P04259 | 60 kDa | 4 |
| 252 | Trophoblast glycoprotein | TPBG_HUMAN | Q13641 | 46 kDa | 4 |

**Table-S2**

| **S.No** | **Gene Symbol** | **Gene Name** | **Entrez Gene ID** |
| --- | --- | --- | --- |
| 1 | COL1A1 | collagen type I alpha 1 chain | 1277 |
| 2 | COL1A2 | collagen type I alpha 2 chain | 1278 |
| 3 | COL6A1 | collagen type VI alpha 1 chain | 1291 |
| 4 | COL6A2 | collagen type VI alpha 2 chain | 1292 |
| 5 | COL6A3 | collagen type VI alpha 3 chain | 1293 |
| 6 | COL12A1 | collagen type XII alpha 1 chain | 1303 |
| 7 | FLNA | filamin A | 2316 |
| 8 | FLNB | filamin B | 2317 |
| 9 | FN1 | fibronectin 1 | 2335 |
| 10 | ITGA5 | integrin subunit alpha 5 | 3678 |
| 11 | ITGAV | integrin subunit alpha V | 3685 |
| 12 | ITGB1 | integrin subunit beta 1 | 3688 |
| 13 | LAMC1 | laminin subunit gamma 1 | 3915 |
| 14 | TLN1 | talin 1 | 7094 |
| 15 | COL14A1 | collagen type XIV alpha 1 chain | 7373 |
| 16 | VCL | vinculin | 7414 |
| 17 | ACTN4 | actinin alpha 4 | 81 |
| 18 | ACTN1 | actinin alpha 1 | 87 |
| 19 | ITGB3 | Integrin beta 3 | 77 |

**Table-S3**

| **S.No** | **Gene Symbol** | **Gene Name** | **Entrez Gene ID** |
| --- | --- | --- | --- |
| 1 | COL6A1 | collagen type VI alpha 1 chain | 1291 |
| 2 | COL6A2 | collagen type VI alpha 2 chain | 1292 |
| 3 | COL6A3 | collagen type VI alpha 3 chain | 1293 |
| 4 | COL12A1 | collagen type XII alpha 1 chain | 1303 |
| 5 | ITGB1 | integrin subunit beta 1 | 3688 |
| 6 | MYH9 | myosin heavy chain 9 | 4627 |
| 7 | MYH10 | myosin heavy chain 10 | 4628 |
| 8 | COL14A1 | collagen type XIV alpha 1 chain | 7373 |
| 9 | CAMK2D | calcium/calmodulin dependent protein kinase II delta | 817 |

**Table-S4**

| **S.No** | **Gene Symbol** | **Gene Name** | **Entrez Gene ID** |
| --- | --- | --- | --- |
| 1 | CPQ | carboxypeptidase Q | 10404 |
| 2 | MYL12A | myosin light chain 12A | 10627 |
| 3 | CLIC1 | chloride intracellular channel 1 | 1192 |
| 4 | COL1A1 | collagen type I alpha 1 chain | 1277 |
| 5 | COL1A2 | collagen type I alpha 2 chain | 1278 |
| 6 | EGFR | epidermal growth factor receptor | 1956 |
| 7 | FAP | fibroblast activation protein alpha | 2191 |
| 8 | FLNA | filamin A | 2316 |
| 9 | FN1 | fibronectin 1 | 2335 |
| 10 | MYOF | myoferlin | 26509 |
| 11 | GSN | gelsolin | 2934 |
| 12 | ANXA1 | annexin A1 | 301 |
| 13 | ANXA2 | annexin A2 | 302 |
| 14 | ANXA5 | annexin A5 | 308 |
| 15 | EHD2 | EH domain containing 2 | 30846 |
| 16 | HSPB1 | heat shock protein family B (small) member 1 | 3315 |
| 17 | ITGA5 | integrin subunit alpha 5 | 3678 |
| 18 | ITGB3 | integrin subunit beta 3 | 3690 |
| 19 | KRT1 | keratin 1 | 3848 |
| 20 | MYH9 | myosin heavy chain 9 | 4627 |
| 21 | MYH10 | myosin heavy chain 10 | 4628 |
| 22 | P2RX4 | purinergic receptor P2X 4 | 5025 |
| 23 | PDGFRB | platelet derived growth factor receptor beta | 5159 |
| 24 | SERPINE2 | serpin family E member 2 | 5270 |
| 25 | PKM | pyruvate kinase, muscle | 5315 |
| 26 | PRCP | prolylcarboxypeptidase | 5547 |
| 27 | S100A8 | S100 calcium binding protein A8 | 6279 |
| 28 | S100A9 | S100 calcium binding protein A9 | 6280 |
| 29 | THBS1 | thrombospondin 1 | 7057 |
| 30 | C1QBP | complement C1q binding protein | 708 |
| 31 | TLN1 | talin 1 | 7094 |
| 32 | VCL | vinculin | 7414 |
| 33 | ACTN1 | actinin alpha 1 | 87 |
| 34 | CD9 | CD9 molecule | 928 |
| 35 | CD36 | CD36 molecule | 948 |
| 36 | CD59 | CD59 molecule | 966 |

**Table-S5**

| **S.No** | **Gene Symbol** | **Gene Name** | **Entrez Gene ID** |
| --- | --- | --- | --- |
| 1 | PRDX4 | peroxiredoxin 4 | 10549 |
| 2 | PRDX3 | peroxiredoxin 3 | 10935 |
| 3 | ALB | albumin | 213 |
| 4 | GSR | glutathione-disulfide reductase | 2936 |
| 5 | GSTM1 | glutathione S-transferase mu 1 | 2944 |
| 6 | GSTM2 | glutathione S-transferase mu 2 | 2946 |
| 7 | GSTP1 | glutathione S-transferase pi 1 | 2950 |
| 8 | PRDX1 | peroxiredoxin 1 | 5052 |
| 9 | S100A9 | S100 calcium binding protein A9 | 6280 |
| 10 | SOD1 | superoxide dismutase 1 | 6647 |
| 11 | PRDX2 | peroxiredoxin 2 | 7001 |
| 12 | TXN | thioredoxin | 7295 |
| 13 | TXNRD1 | thioredoxin reductase 1 | 7296 |
| 14 | TXNDC17 | thioredoxin domain containing 17 | 84817 |
| 15 | GSTO1 | glutathione S-transferase omega 1 | 9446 |
| 16 | CD36 | CD36 molecule | 948 |
| 17 | PRDX6 | peroxiredoxin 6 | 9588 |

**Figure-S1**


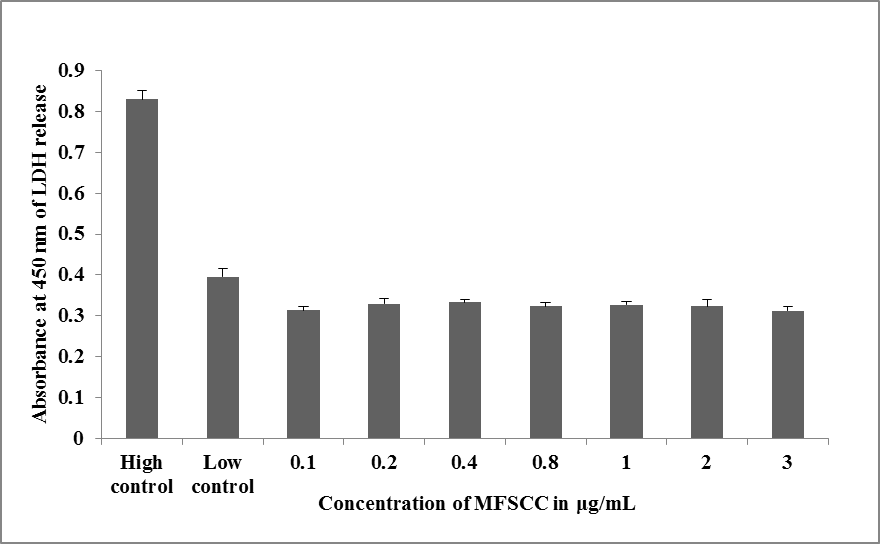

Supplement: Supplementary Materials — Table S1: list of total proteins from membrane-free stem cell component (MFSCC) using Nano-LS/MS analysis. Table S2: list of proteins from MFSCC that are involved in the integrin signaling pathway. Table S3: list of proteins from MFSCC that are involved in the inflammatory-mediated pathway. Table S4: list of proteins from MFSCC that are involved in the wound healing pathway. Table S5: list of proteins from MFSCC that are involved in the cellular detoxification pathway. Figure S1: cytotoxic effect of MFSCC on RAW264.7 by LDH assay. RAW264.7 macrophage cells were treated with MFSCC at the indicated concentration of 0.1 to 3 μg/mL for 24 h. Cell cytotoxicity assay kit (Duzen Bio Co., Ltd., Guro-gu, Republic of Korea) was used, according to the manufacturer's instructions. [file 4683272.f1.docx]
